# Transfer of physical understanding in a non-tool-using parrot

**DOI:** 10.1007/s10071-016-1031-0

**Published:** 2016-09-17

**Authors:** Jayden O. van Horik, Nathan J. Emery

**Affiliations:** 1 School of Biological and Chemical Sciences, Queen Mary University of London, London, E1 4NS UK; 2 Centre for Research in Animal Behaviour, College of Life and Environmental Sciences, University of Exeter, Exeter, EX4 4QG UK

**Keywords:** Physical cognition, Parrots, Means-end, Causal reasoning, Behavioural flexibility, Tool-use, Trap-Gaps

## Abstract

Physical cognition has generally been assessed in tool-using species that possess a relatively large brain size, such as corvids and apes. Parrots, like corvids and apes, also have large relative brain sizes, yet although parrots rarely use tools in the wild, growing evidence suggests comparable performances on physical cognition tasks. It is, however, unclear whether success on such tasks is facilitated by previous experience and training procedures. We therefore investigated physical comprehension of object relationships in two non-tool-using species of captive neotropical parrots on a new means-end paradigm, the Trap-Gaps task, using unfamiliar materials and modified training procedures that precluded procedural cues. Red-shouldered macaws (*Diopsittaca nobilis*) and black-headed caiques (*Pionites melanocephala*) were presented with an initial task that required them to discriminate between pulling food trays through gaps while attending to the respective width of the gaps and size of the trays. Subjects were then presented with a novel, but functionally equivalent, transfer task. Six of eight birds solved the initial task through trial-and-error learning. Four of these six birds solved the transfer task, with one caique demonstrating spontaneous comprehension. These findings suggest that non-tool-using parrots may possess capacities for sophisticated physical cognition by generalising previously learned rules across novel problems.

## Introduction

The comprehension of object relationships has typically been assessed using both vertical string-pulling and horizontal means-end problems. Vertical string-pulling tasks typically require subjects to discriminate between strings that are either connected, or disconnected, to an otherwise out-of-reach reward. Subjects then perform potentially novel and coordinated motor actions to pull up the string to obtain the reward, and their performances may improve with experience. While numerous species of birds have been tested on vertical string-pulling tasks (Jacobs and Osvath 2015), only budgerigars, *Melopsittacus undulates* (Ducker and Rensch 1977), parrots (Krasheninnikova et al. 2013; Schuck-Paim et al. 2009; Werdenich and Huber 2006) and corvids (Heinrich 1995; Heinrich and Bugnyar 2005) show an ability to rapidly, if not spontaneously, solve more complicated configurations of this task, such as correctly discriminating between rewarded, rather than unrewarded crossed strings and broken strings, as well as discriminating between pulling strings based on the perceived effort required to retrieve a reward (Pfuhl 2012).

Horizontal means-end discrimination tasks, by contrast, are often used to assess physical cognition in primates, typically by presenting subjects with a binary choice between a functional and a non-functional tool or a platform, with rewards placed at the distal ends of each option (Hauser et al. 1999; Povinelli 2000; Yamazaki et al. 2011). However, a variety of species, such as dogs (Müller et al. 2014) and elephants (Irie-Sugimoto et al. 2008), also show capacities to solve this problem. While non-human great apes essentially demonstrate spontaneous comprehension of horizontal means-end problems (Herrmann et al. 2008; Mulcahy et al. 2013), yellow-crowned parakeets, *Cyanoramphus auriceps* (Funk 2002), blue-fronted Amazons, *Amazona aestival* (De Mendonca-Furtado and Ottoni 2008), and pigeons, *Columba livia* (Schmidt and Cook 2006), are less adept. By contrast, ravens, *Corvus corax*, and crows, *C. corone, C. cornix*, are capable of solving more complicated crossed support tasks (Albiach-Serrano et al. 2012; Bagotskaya et al. 2012), yet these corvids, in contrast to great apes, *Gorilla gorilla, Pan paniscus, P. troglodytes, Pongo abelii*, do not appear to comprehend the causal principles of such problems (Albiach-Serrano et al. 2012; Bagotskaya et al. 2012). The most convincing evidence that parrots are capable of comprehending the causal principles underlying means-end problems has been demonstrated by keas, *Nestor notabilis*, in which one of six individuals showed spontaneous success by pulling a continuous, rather than disrupted, wooden slat to retrieve an otherwise out-of-reach food reward (Auersperg et al. 2009). Similar performances were also found in one of four black-headed caiques, *Pionites melanocephala*, but not among four red-shouldered macaws *Diopsittaca nobilis*, presented with continuous or disrupted strips of cloth that were either connected or disconnected to a reward (van Horik and Emery unpublished observations).

Trap-Table (Povinelli 2000), Two-Trap-Tube (Seed et al. 2006) and Trap-Barrier (Martin-Ordas et al. 2012) tasks share similar functional properties. In these tasks, the subject must avoid a trap or barrier to access a food reward. However, only a few individuals have been successful on these problems. In species that naturally use tools in the wild, such as chimpanzees, Pan troglodytes, individuals rarely succeed at the Trap-Table task (Povinelli 2000) but show greater success on modified Trap-Table problems when they could choose where to insert a single tool on a binary problem, rather than when choosing between two previously inserted tools (Girndt et al. 2008) and when using their fingers, rather than tools, to move a reward on a similar Two-Trap-Box task (Seed et al. 2009). However, species that do not naturally use tools in the wild, such as hoolock gibbons, Hylobates hoolock (Cunningham et al. 2006), and vervet monkeys, Chlorocebus pygerythrus (Santos et al. 2006), also show capacities to solve the Trap-Table task, although tamarins, Saguinus Oedipus (Santos et al. 2006), and capuchin monkeys, Cebus apaella (Fujita et al. 2003), failed. Similar findings have been found among tool-using and non-tool-using corvids on a Trap-Tube task (Seed et al. 2006; Tebbich et al. 2007; Taylor et al. 2009), whereas numerous species of parrots fail similar tasks (Liedtke et al. 2010).

Due to their functional similarities, Trap tasks have also been used to assess the ability to transfer previously learned information across novel problems. Great apes, however, failed to generalise information across the Trap-Table and Trap-Tube problems (Martin-Ordas et al. 2008), although in a similar task, apes previously exposed to trap or barrier platforms outperformed subjects that initially received a non-obstacle platform (Martin-Ordas et al. 2012). Only New Caledonian crows, *Corvus moneduloides*, that previously solved a Two-Trap-Tube task could solve a Trap-Table transfer task, while those that failed to solve the Two-Trap-Tube task also failed the Trap-Table task (Taylor et al. 2009). However, as presentation order of these tasks were not counterbalanced across subjects, it remains difficult to interpret whether successful birds solved these problems by generalising causally relevant principles across the different tasks.

Assessing different capacities to comprehend the underlying causal relationships between objects may help reveal the selection pressures that drive the evolution of cognition (van Horik et al. 2012). The majority of physical cognition tasks are, however, based around tool-using paradigms, possibly because research on physical cognition has typically focused on species that frequently use tools in the wild, such as the great apes (Povinelli 2000; Tomasello and Call 1997). Yet species that do not naturally use tools also show capacities for physical cognition when tested in captivity. For example, vervet monkeys which do not regularly use tools in the wild outperformed tool-using chimpanzees on a Trap-Table task (Povinelli 2000; Santos et al. 2006). Moreover, corvids which do not use tools in the wild, such as rooks (Bird and Emery 2009a, b; Seed et al. 2006), show capacities for physical cognition that rival corvids that do frequently use tools in the wild, such as New Caledonian crows (Taylor et al. 2009; Weir et al. 2002) and possibly great apes (Hanus et al. 2011; Mendes et al. 2007). As such, habitual tool users do not currently appear to possess greater capacities for physical cognition than non-tool-using species.

Successful performances of both tool-using and non-tool-using species on some means-end tasks may, however, be facilitated by simple perceptual cues experienced during training. As such, subjects may solve these tasks without possessing any causal understanding of the problem. In some studies for example (Auersperg et al. 2009; Cunningham et al. 2006; Irie-Sugimoto et al. 2008), subjects were initially provided with training on a continuous surface (i.e. without a trap), or with continuous (i.e. unbroken) materials, before being presented with traps or non-functional materials that are disconnected to the reward. Hence, individuals may solve the task through previously learnt associations between the reward and the continuous surface or materials, rather than by understanding the causal properties of traps and connectivity (but see Povinelli 2000). Paradigms that preclude the use of such perceptual cues, by using novel transfer tasks that manipulate causal rules associated with object relationships, may therefore better illuminate differences in physical cognition and facilitate more accurate comparisons across species.

Due to their large relative brain sizes (Iwaniuk et al. 2005) and other socio-ecological traits shared with corvids and great apes, such as complex sociality (Hobson et al. 2014), dexterity required for omnivorous extractive foraging, and protracted developmental period and lifespan, parrots may reveal similar capacities for physical cognition (van Horik and Emery 2011; van Horik et al. 2012). In the current study, we investigate whether two species of non-tool-using parrots show capacities for physical cognition when tested on a means-end problem that lacks similar perceptual cues experienced during training. To do this, we introduce a new means-end transfer paradigm that alleviates the training biases which may promote learned perceptual rules and limit interpretations drawn from previous studies. We designed a Trap-Gaps problem to investigate whether red-shouldered macaws and black-headed caiques can generalise learned rules about object relationships across novel problems. We assess whether subjects can discriminate between pulling food trays through gaps while attending to the respective width of the gaps and the size of the trays. Subjects were presented with two tasks in a counterbalanced order: one in which the size of the gaps varied, but the size of the trays remained constant, and another task where the size of the trays varied, but the size of the gaps remained constant. Hence, subjects could learn about the relationships between objects through trial-and-error associative learning in the first task, but potentially solve the second task spontaneously if they transferred these causally relevant principles across the different problems.

## Methods

### Subjects

Four red-shouldered macaws: No. 2, No. 4, No. 5 and No. 8, and four black-headed caiques: Green, Gold, Purple and Red (hereafter macaws and caiques), participated in this study. All subjects, with the exception of No. 4, were male. Subjects were hand-reared and each species were from at least two different clutches. Individuals from each species were 33 months old when tested and housed according to species in indoor aviaries (2 m^3^) under identical conditions.

Subjects had prior experience with a number of tasks employing object manipulation, including removing food hidden under different coloured lids (van Horik and Emery unpublished data). At nine months of age, subjects were also presented with 100 trials on a means-end connected task, where they were required to discriminate between pulling vertical pieces of white cloth (25 mm wide × 160 mm long) that were either continuous, and hence connected to a distal reward, or separated by a 15-mm gap. One caique (Gold) made 9 correct choices on his first 10 trails (van Horik and Emery unpublished data). Subjects, however, had no prior experience with pulling trays through gaps, or the any of the materials, colours, strings or trays used in the current experiment. Both species were raised under identical conditions and provided with equal experiences. Food and water were provided that ad libitum and subjects’ participation was voluntary, i.e. they were not forced to engage with the test apparatus but did so of their own volition.

## Materials

- *Training task*

**Fig. 1:**
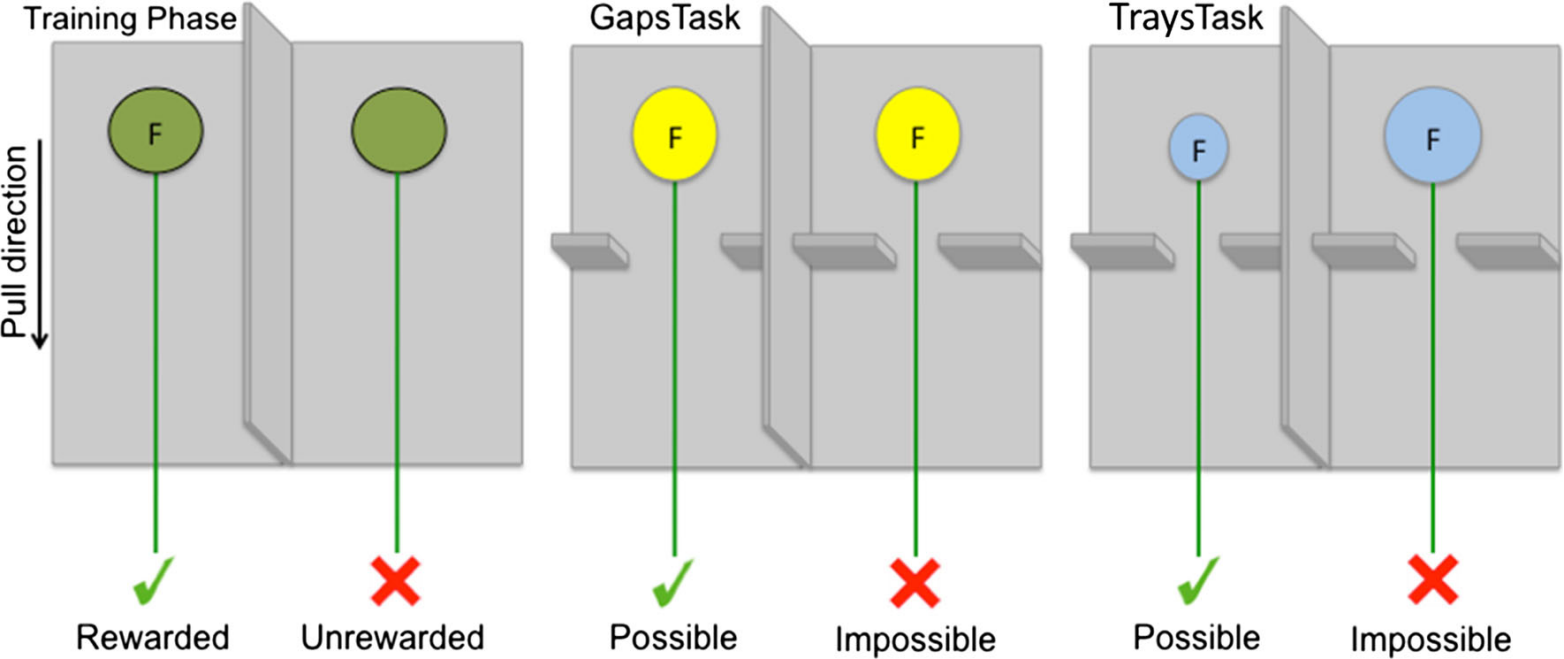
Trap-Gaps Training and Test apparatus (not to scale). Food-reward trays (F) can be pulled towards the subject via a green string. Subjects commence with the training phase then proceed with the Trays or Gaps tasks in a counterbalanced order

Subjects were presented with two identical opaque green trays (55 mm diameter × 20 mm deep), each attached to a 200-mm-long green string and positioned out of the subjects’ reach at the distal end of a partitioned A4 letter tray (Fig. 1). The letter tray was fixed to the outside of the testing arena, and only one tray was baited with a clearly visible food reward. While the trays were opaque, they were wide and shallow. Hence, the rewards were not concealed and the contents of each tray remained clearly visible to subjects at all times. Either tray could be retrieved by pulling their respective string, but subjects were required to discriminate between pulling strings that were attached to a rewarded rather than an unrewarded tray. Subjects were not presented with gaps during training trials.

(b) *Gaps task*

Two identical opaque yellow trays (60 mm diameter × 20 mm deep), both baited with equal quantities of clearly visible food rewards, were positioned in the same location as above. A 200-mm-long green string was attached to each tray, allowing the trays to be pulled towards the cage (Fig. 1). To retrieve the food reward, subjects were required to discriminate between two different sized gaps. One gap (70 mm wide × 50 mm high) was large enough to allow the passage of the tray, whereas the other gap (50 mm wide × 50 mm high) restricted access to the tray. Both gaps were positioned 170 mm from the subject, with the trays placed approximately 30 mm behind the gaps.

(c) *Trays task*

Two gaps of equal size (65 mm wide × 50 mm high) were positioned as above. Two baited blue trays, one large (75 mm diameter, 20 mm deep) and one small (55 mm diameter × 20 mm deep), were baited as above. Apart from their size, both trays were identical. The gaps allowed passage of the small tray but restricted access to the large tray.

### Procedure

Subjects were tested individually in a familiar aviary that was visually isolated from other individuals. Subjects were initially presented with the training apparatus, requiring them to discriminate between rewarded and non-rewarded trays, and then subsequently tested on the Trays and Gaps tasks (Fig. 1). All trays were opaque, but their contents clearly visible to the subjects. Each apparatus was positioned outside of the aviary, behind a clear Plexiglas barrier, with the strings initially out of the subject’s reach. After baiting the trays with a clearly visible food reward of crushed orange Lafeber Nutri-Berries™, the experimenter then waited until the subject was within 10 cm of the apparatus before administering a 5-s delay to allow the subject time to view, but not access, the baited tray. Both strings were then simultaneously presented, so that they protruded under the Plexiglas barrier and were within the subjects reach. Subjects were only allowed one attempt per trial to pull a string and were considered to have made a correct choice if they retrieved the reward. The first sting that the subject picked up, with either their bill or foot, was scored as a choice. Subjects always retrieved the rewarded tray after pulling the correct string, whereas the incorrect (but still baited) tray always remained inaccessible. The apparatus was removed after subjects pulled either a correct or incorrect string and rebaited for subsequent trials. Subjects received a maximum of 10 trials per day. The location of the accessible reward was pseudo-randomised so that it did not occur on the same side for more than two consecutive trials. Trials were recorded with a digital camcorder (JVC Everio, Model No. GZ-MG645BEK, Malaysia), and the outcome of each trial was coded live via the camcorder’s monitor. During trials, the experimenter’s hands were placed behind his back. A random selection of 10 % of all trials (*N* = 126) were independently recoded by a naïve observer (A. Hulatt) to determine inter-observer reliability ratings of videos, revealing 100 % agreement.

- *Training: discriminating between rewarded and unrewarded trays*

To proceed to the test condition, subjects were required to choose the baited tray for 7 consecutive trials in one block of 10 trials. After reaching criterion, subjects proceeded to the test condition on the following day. Subjects were considered to have developed a side bias if they chose the same side in six or more trials. To correct for side biases, the non-preferred side was consistently baited until the subject retrieved the reward over two consecutive trials. The presentation order then resumed to its original pseudo-randomised configuration. Errors from side bias corrective trials were included in the training analyses.

(b) *Learning and transfer*

After subjects mastered the training phase, they were provided with the Gaps and Trays tasks in a counterbalanced order. That is, after training to discriminate rewarded from non-rewarded trays, half of the test subjects proceeded with the Trays task, whereas the other half proceeded with the Gaps task. Individuals from each species were pseudo-randomly assigned to each task order. The first task that each subject experienced was considered an “initial learning task”, in which subjects could learn the relationship between the trays and barriers. Subjects were then required to reach a predetermined learning criterion of either 7 consecutive, or 9 out of 10, correct choices in one block of 10 trials before participating in the subsequent “transfer” task. Two learning criterions were used in an attempt to maintain subjects’ interest in interacting with each apparatus. Side biases were corrected, as above, using the training apparatus. Corrective trials, using the training apparatus, were not included in subsequent analyses. Testing ceased if a subject failed to reach criteria on their initial task within 200 trials, or if subjects failed to reach the transfer task criteria with 100 trials. To determine whether subjects retained previously learned information, subjects that reached criteria on each task were subsequently re-tested with one additional block of 10 trials on their initial task.

### Statistical analysis

We used Mann–Whitney *U* tests to compare the number of errors and trials that each group made before successfully reaching the training criterion. Exact tests were reported following Mundry and Fischer (1998) to accommodate analysis of our small sample size. Retrospectively, through processes of enumeration, we determined a probability of *P* = 0.02 that a given subject would meet the above learning criteria within one 10-trial session. To do this, we summed the number of possible successful combinations that could be made in one 10-trial session (i.e. 20 combinations of reaching at least seven consecutive correct choices in 10 trials, plus four combinations of nine correct choices out of 10 trials) and divided this by the total number of possible choices (i.e. 24 successful combinations out of 1024 possible choices, i.e. each of the ten trials in a given session presents 2 options). As the learning criterion was assessed repeatedly across sessions, we assigned each subject with a cumulative probability of success to account for the increased likelihood of success due to multiple testing. Each subject’s probability of success was therefore weighted by the number of sessions it required to either reach criterion, or by the total number of sessions a subject participated in before testing ceased (*cf* Grant 1946). Cumulative probabilities were then used to determine Chi-squared values for each successful subject. The summed Chi-squared values were then tested against the summed degrees of freedom (all 1-tailed) using Fishers methods (Sokal and Rohlf 1995; pp 734) and used to determine an overall probability that the successful performances of subjects differed significantly from chance.

## Results

### Training: discriminating between rewarded and unrewarded trays

Five of eight subjects chose the correct side on their first trial of the training task. There were no significant between species differences in the number of errors made (macaws median = 10, range = 4–23; caiques median = 5, range = 0–17; Mann–Whitney *U* test, *U* = 5.0, *N*_*1*_ = *4, N*_*2*_ = *4*, *P* = 0.49), or number of trials (macaws median = 29, range = 18–77; caiques median = 23, range = 7–49; Mann–Whitney *U* test, *U* = 7, *N*_*1*_ = *4, N*_*2*_ = *4*, *P* = 0.89), to reach the initial training criterion. Two individuals from each species were therefore randomly assigned to receive trials commencing with either task. There were no significant differences in errors (Trays task median = 11, range = 5–16; Gaps task median = 5, range = 0–13: Mann–Whitney *U* test, *U* = 2.5, *N*_*1*_ = *4, N*_*2*_ = *4*, *P* = 0.14), or trials (Trays task median = 38, range = 18–77; Gaps task median = 19, range = 7–40; Mann–Whitney *U* test, *U* = 3.5, *N*_*1*_ = *4, N*_*2*_ = *4*, *P* = 0.20), to reach the training criterion between these two groups. However, due to limitations of a small sample size these comparisons are likely to suffer from low power, and hence, any differences between these groups may be difficult to reveal. During training, two macaws, No. 2 and No. 5, developed side biases in sessions 3 and 1, respectively, which were included in subsequent analyses of training performances.

### Learning and transfer

Four of eight subjects (No. 4, No. 5, No. 8 and Purple) chose the correct side on their first trial in the initial task, while five (No. 2, No. 4, No. 5, Gold and Red) of six subjects were correct on their first transfer task trial. Although the cumulative probability of success in Task 1 failed to reach statistical significance (*χ* ^2^ = 25.86, DF = 16, *P* = 0.06), suggesting that the initial learning criteria were not rigorous enough, subjects’ performances differed significantly from chance in Task 2 (*χ* ^2^ = 26.67, DF = 12, *P* = 0.009) and in Task 1b (*χ* ^2^ = 30.03, DF = 8, *P* = 0.0002) when they were retested on a further 10 trials of their initial learning task. Subjects frequently developed side biases throughout testing. Side biases were formed during the following sessions: No. 2: Task 1 Session 1, Task 2 Session 3; No. 4: Task 1 Sessions 2, 3, 4; Green: Task 1 Sessions 1, 2, 6, 8, 9, 14; Red: Task 1 Sessions 2, 7; No. 5: Task 1 Sessions 1, 2, 4; No. 8: Task 1 Sessions 1, 2, 3, 4, 5, 6, 9, 12, 14, 17, 21, 22; Gold: Task 1 Sessions 1, 2, Task 2 Sessions 2, 4; and Purple: Task 1 Sessions 3, 9, 10, 12, 16.

- *Trays task*

All four subjects that commenced with the Trays task reached the criterion to transfer to the second task (median errors: 38.50, range = 20–64; median trials: 90.00, range 45–134; Table 1). Only No. 4 and Red reached our criteria in the transfer condition. While No. 4 demonstrated similar performances on both tasks, when re-tested on the initial task this subject immediately reached criteria by choosing the correct tray on all 10 trials.

**Table 1:** Number of errors and trials to reach criterion for the training phase and groups commencing with the Trays task and then transferring to the Gaps task and vice versa

	Training		Learning		Transfer		Retest	
Subjects	Food	Discrimination	Task 1:	Trays	Task 2:	Gaps	Task 1b:	Trays
	Errors	Trials	Errors	Trials	Errors	Trials	Errors	Trials
No. 2	23	77	20	47	+33	+100	N/A	N/A
No. 4	6	18	20	45	21	68	0*	10
Green	17	49	64	134	+51	+100	N/A	N/A
Red	5	27	57	133	0*	9	0*	10

			Task 1:	Gaps	Task 2:	Trays	Task 1b:	Gaps
No. 5	13	40	24	67	11	38	5	10
No. 8	4	18	+90	+200	N/A	N/A	N/A	N/A
Gold	0	7	10	32	20	46	3	10
Purple	5	19	+93	+200	N/A	N/A	N/A	N/A

Task 1b shows the number of errors that subjects made when retested on a further 10 trials of their initial task. Cells denoted by a “+” indicate that individuals failed to reach criterion within the corresponding number of errors; “N/A” indicates that subjects were not presented with the transfer task; and “*” indicates that subjects performed significantly above chance within 10 trials (*P* < 0.01). Note that Red participated in 10 trials in Task 2, only making its first error on the 10th trial

Only one subject’s performance (Red) suggested capacities to generalise previously learned relationships across novel, but functionally equivalent, problems. Red spontaneously reached our learning criteria on the transfer task, choosing the correct side for its first nine out of 10 trials. Red again reached criteria when retested on the first task, successfully choosing the correct tray on all 10 trials.

(b) *Gaps task*

Only No. 5 and Gold reached criteria on the Gaps task (median errors: 17.00, range = 10–24; median trials: 49.50, range 32–67; Table 1). Two birds, No. 8 and Purple, failed to reach the initial task criteria within 200 trials and were therefore not presented with the transfer task. Although Gold rapidly reached criteria on its first task, it made twice as many errors on the transfer task. While No. 5 reached criteria with fewer errors than the initial task, both No. 5 and Gold failed to reach criteria when retested on a further 10 trials of the initial Gaps task.

## Discussion

Red-shouldered macaws and black-headed caiques successfully learned to solve a means-end problem that required an ability to discriminate between pulling baited trays through gaps, while attending to the respective width of the gaps or size of the trays. The performances of one subject, when presented with a subsequent transfer task, may also suggest that some parrots are capable of retaining causally relevant information about object relations and then using this experience to generalise information across a novel, but functionally equivalent, problems.

 While all subjects learned to discriminate rewarded from unrewarded trays during the training phase, of the eight subjects tested, three macaws and three caiques learned to solve their initial presentation of the Trap-Gaps task. Yet, subjects’ performances varied considerably, suggesting that the ability to discriminate between the size relationships of the trays and gaps required trial-and-error experience and may be a particularly difficult problem for these parrots to comprehend. While all four subjects that commenced with the Trays task learned to solve the problem, only two subjects solved the initial presentation of the Gaps task. As such, the ability to discriminate between different sized gaps may be more challenging than discriminating between different sized trays. These findings may, however, be due to the experience that subjects received during training, which required attending to rewarded and unrewarded trays in the absence of gaps. Hence, the different sized gaps may have been a less salient feature of the task than the different sized trays. These findings may also explain why many species struggle with the Trap-Tube and Trap-Table tasks as they have to attend to a gap removed from the goal object. In contrast, to solve the Trays task subjects have to attend to the goal object itself. The colour of the trays and the reward, crushed orange Nutriberries, may have also been more conspicuous in the Trays task, where the trays were blue, rather than in the Gaps task where yellow trays were used. However, the contents of each tray were clearly visible and subjects rapidly succeeded in discriminating rewarded from unrewarded green trays. While the trays differed in colour between the training and test conditions, the similar size of the training task trays and the small (accessible) Trays task tray may have also facilitated subjects’ performances on this task. Future studies may therefore benefit by increasing the size differences between the trays in each condition.

To test whether subjects solved the initial task through perceptual cues, or whether they showed a causal understanding of object relations, we presented the six birds that successfully learnt to solve the initial task, three macaws and three caiques, with a novel, but functionally equivalent, transfer task. Four of six subjects reached criterion within 100 trials on the transfer task. One macaw made less than half as many errors on the transfer task than the initial task and one caique spontaneously reached criterion without making any errors. The remaining two birds took considerably longer to reach criteria on the transfer task than compared to their initial performances. As subjects’ overall performances on the initial task failed to differ significantly from chance, it remains possible that those successful individuals, that reached our predetermined learning criteria, did so without adequately learning the affordances of the problem. Subjects’ performances on the transfer task may therefore have improved by introducing a more stringent learning criterion during the initial tasks. While spontaneous success on the transfer task may suggest that at least one subject possessed a causal understanding of the relations between trays and gaps, it remains possible that subjects’ performances on the transfer task were facilitated by simple perceptual cues learnt during their exposure to the first task. For example, subjects might learn that trays partially occluded by the barrier were inaccessible. While the apparatus was presented in full view, and subjects could clearly see behind the 50-mm-high barriers, this relatively simple associative rule, to choose the fully visible tray, may therefore be sufficient to solve this task. Future studies may therefore consider manipulating such perspectives to include conditions in which the larger tray, or the tray behind the small gap, is placed at further distances to keep the visual cues available to the subjects comparable.

The fact that most subjects learnt to solve the initial task, but failed to rapidly solve the transfer task suggests that subjects may have learnt to attend to only one dimension of each problem, i.e. the size of the trays or the size of the gaps, rather than the combined relation between the trays and gaps. As such, rather than generalising rules across tasks, subjects may have been confronted with an object-relation reversal task, which may require additional trials to unlearn the first rule and then subsequently learn the new rule. After learning each rule independently, (a) to choose the small tray and (b) to choose the large gap, the poor performances of two subjects on the retest of Task 1 (i.e. Task 1b) may therefore suggest that they were attempting to generalise a new rule to solve the initial problem, rather than reverting to the initially learnt rule. Unlike the numerous species of parrots that failed to solve Trap-Tube problems (Liedtke et al. 2010), the macaws and caiques in the current study, like rooks (Seed et al. 2006), may have successfully solved their initial task by applying a procedural rule based on an arbitrary cue, such as attending to the size of the trays in relation to the size of the gaps. The ability to recall learned procedural rules may, however, also be a particularly efficient approach to solving repeatedly encountered problems. As such, two of the four subjects re-tested on a further ten trials of their initial task solved the problem without making any errors.

Red-shouldered macaws and black-headed caiques naturally forage in the forest canopy (Juniper and Parr 2003), and an understanding of distal object relations, for example when pulling branchlets containing berries or seeds towards themselves, may provide a selective advantage if it enhances an individual’s foraging efficiency. Evidence to suggest that parrots possess capacities for complex physical cognition, comparable to other relatively large brained birds and mammals, is growing (van Horik et al. 2012). As such, parrots have been observed to spontaneously make stick-tools in captivity (Auersperg et al. 2012) and may comprehend physical concepts of objects such as functionality and connectivity (Auersperg et al. 2009) due to their motivation to manipulate and explore inanimate objects (Auersperg et al. 2015). In contrast to the unsuccessful performances of parrots on some physical cognition tasks (Liedtke et al. 2010), findings from the current study bolster support for complex physical cognition among non-tool-using parrots. While the current study is constrained by a small sample size and possible procedural concerns, our findings suggest that the Trap-Gaps paradigm may be a particularly useful approach for assessing physical cognition among a broad variety of both tool-using and non-tool-using species. Moreover, the Trap-Gaps paradigm refines previous means-end and Trap-Table paradigms, as the training procedures do not confound interpretations of the experimental trials by previously rewarding a continuous string or platform.

By removing some of the perceptually learned cues that subjects experience during training procedures in previous mean-end tasks, the Trap-Gaps transfer paradigm, presented in the current study, may provide an additional method to clarify whether tool-using and non-tool-using species with large relative brain sizes possess similar capacities for physical cognition. Findings from the current study suggest that parrots, like corvids (Seed et al. 2006; Taylor et al. 2009) and great apes (Martin-Ordas et al. 2008, 2012), may possess capacities to solve complicated means-end problems by generalising previously learned experiences to help solve novel problems.
